# Bioactive glass doped with noble metal nanoparticles for bone regeneration: *in vitro* kinetics and proliferative impact on human bone cell line†

**DOI:** 10.1039/d1ra03876a

**Published:** 2021-07-25

**Authors:** Amany A. Mostafa, Mayyada M. H. El-Sayed, Ahmed N. Emam, Ahmed A. Abd-Rabou, Reham M. Dawood, Hassane Oudadesse

**Affiliations:** Refractories, Ceramics and Building Materials Department (Biomaterials Group), National Research Centre (NRC) El Bohouth St., Dokki 12622 Cairo Egypt amani.mostafa@gmail.com aa.monem@nrc.sci.eg; Nanomedicine & Tissue Engineering Lab., Medical Research Center of Excellence (MRCE), NRC Egypt; Chemistry Department, School of Sciences and Engineering, American University in Cairo AUC Avenue New Cairo 11835 Egypt Mayyada@aucegypt.edu; Hormones Department, Medical Research Division, National Research Centre Dokki Giza Egypt; Department of Microbial Biotechnology, Genetic Engineering Division, National Research Centre 33 EL Bohouth Street Dokki Giza 12622 Egypt; Universite de Rennes 1, UMR CNRS 6226 263 Avenue du Général Leclerc 35042 Rennes Cedex France

## Abstract

This work investigates the bioactivity of novel silver-doped (BG-Ag) and gold-doped (BG-Au) quaternary 46S6 bioactive glasses synthesized *via* a semi-solid-state technique. A pseudo-second-order kinetic model successfully predicted the *in vitro* uptake kinetic profiles of the initial ion-exchange release of Ca in simulated body fluid, the subsequent Si release, and finally, the adsorption of Ca and P onto the bioactive glasses. Doping with silver nanoparticles enhanced the rate of P uptake by up to approximately 90%; whereas doping with gold nanoparticles improved Ca and P uptake rates by up to about 7 and 2 times, respectively; as well as Ca uptake capacity by up to about 19%. The results revealed that the combined effect of Ca and Si release, and possibly the release of silver and gold ions into solution, influenced apatite formation due to their effect on Ca and P uptake rate and capacity. In general, gold-doped bioactive glasses are favoured for enhancing Ca and P uptake rates in addition to Ca uptake capacity. However, silver-doped bioactive glasses being less expensive can be utilized for applications targeting rapid healing. *In vitro* studies showed that BG, BG-Ag and BG-Au had no cytotoxic effects on osteosarcoma MG-63 cells, while they exhibited a remarkable cell proliferation even at low concentration. The prepared bioactive glass doped with noble metal nanoparticles could be potentially used in bone regeneration applications.

## Abstract 2

This work investigates the bioactivity of novel silver-doped (BG-Ag) and gold-doped (BG-Au) quaternary 46S6 bioactive glasses synthesized *via* a semi-solid-state technique.

## Graphical abstract

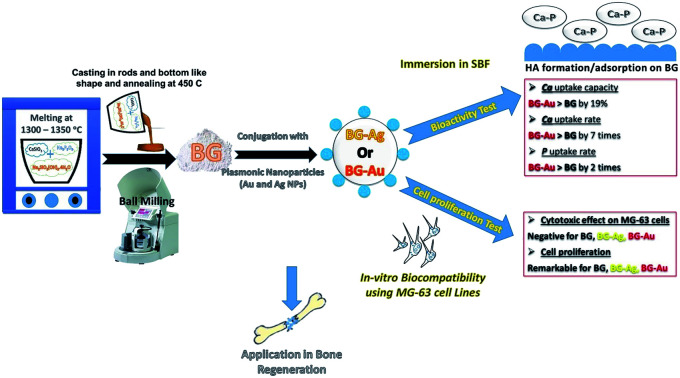

## 1 Introduction

The advance in bone regeneration applications necessitates exploring new biomaterials with specific properties to enhance the formation of apatite while being safe to the human body. Examples of these materials are bio-ceramics, which include bioactive glasses, glass-ceramics, and hydroxyapatites.^1,2^ Bioactive glasses have shown promising biocompatibility, mechanical, and optical properties that render them good candidates for bone implants and tissue engineering. The typical composition of bioactive glass (BG) that was initially discovered consisted mainly of SiO_2_ along with Na_2_O, CaO, and P_2_O_5_. However, other phosphate-based and borate-based bioactive glasses were introduced. Recently, there has been much interest in enhancing the performance of these bioactive glasses and broadening their applications by incorporating ions/particles such as Cu, Zn, Sr, Ag, and Au into the glass network.^3,4^ This can be done through ion-exchange surface processes, and melt-derived or sol–gel derived methods in order to induce osteogenesis as with Zn and Mg, angiogenesis as with Cu and antibacterial activity as with Cu and Ag.^5–8^

Doping 46S6 bioactive glass with 0.1 wt% Zn particles of different sizes reduced the rate of Ca and P adsorption onto the doped glass relative to the non-doped one.^9,10^ However, doping quaternary melt-derived bioactive glass with trace amounts of Mg and Zn enhanced its *in vitro* bioactivity in simulated body fluid (SBF).^4^ Bioactive glasses were also doped with silver to improve their biological activities.^11^ In one study, sol–gel derived nano bioactive glass was doped with 1, 5, and 10 mol% of Ag_2_O, and it was recommended for rapid antibacterial protection due to its fast initial release of Ag ions.^6^ In another study, silver-doped bioactive glass nanoparticles prepared with 1, 3, 5, and 10 mol% of Ag_2_O also exhibited antibacterial properties and recorded a porosity of over 75%.^12^ In addition, bioactive glass (45S5) doped with 0.05 to 0.20 mg l^−1^ of Ag_2_O demonstrated anti-bactericidal activity against *Escherichia coli*, *Pseudomonas aeruginosa*, and *Staphylococcus aureus* microorganisms.^13^ Recently, sol–gel bioglass-based biografts were doped with 5–20% of Ag ions obtained from AgNO_3_ and they showed a potent antibacterial effect on *E. coli* and *S. aureus* at the highest applied concentration of AgNO_3_.^14^ Furthermore, sol–gel bioactive glass was doped with 0.6–4 wt% of Ag_2_O, and the results revealed that the 4% wt composite possessed the highest inhibition power against *C. albicans*, *S. aureus* and *S. mutans*. However, utilizing a tissue conditioner containing this bioactive glass did not promote any antimicrobial properties or cytotoxic effects.^15^ A more recent study reported that surface doping of silica melt bioactive glass with silver ions produced *in vitro* biocompatible materials that can be used for bone self-healing due to their cytocompatibility with human osteoblast progenitor cells. Additionally, their potent antibacterial properties made them effective in preserving the osteoblasts against bacterial infections.^16^ Gold-doped bioactive glasses, as well, showed promising biological activities. The gold-doped 58S bioglass was developed with two different doping ratios of 0.1 and 1% wt to be used in bone reconstruction applications. It also possessed antibacterial properties against the Gram positive *S. aureus*, but had no significant effect on the Gram negative *E. coli*.^17^

The mechanism of hydroxycarbonate apatite formation onto bioactive glass involves many steps, as proposed by Hench, 1996.^18^

It starts with a fast ion-exchange process where Ca^2+^ and Na^+^ cations on the bioactive glass are exchanged with H^+^/H_3_O^+^ ions in solution. This leads to the formation of silanol (Si–OH) groups on the glass surface as the proton replaces the cation bonded to the Si–O. As a result, alkalinity increases in the solution, and OH groups attack the Si–O–Si bonds that form the glass network. Afterward, soluble Si is released in the solution leaving silanol groups on the glass surface. The condensation of these groups forms a silicate layer onto which Ca and P ions are adsorbed.^7,10,19^

In the above studies, doping of the bioactive glass with metal took place *via* an aqueous-phase reaction between the glass and the metal salt or oxide. In the current study, we propose doping the bioactive glass (46S6) with noble metal nanoparticles of silver and gold (*i.e.*, AgNPs and AuNPs, respectively) using a semi-solid-state technique. Herein, we doped the melt-derived bioactive glass (BG) with two concentrations of 0.006 and 0.01 ppm for the AgNPs yielding BG-Ag1 and BG-Ag2 composites, respectively and the same concentrations were also used for AuNPs to obtain BG-Au1 and BG-Au2 composites, respectively.

We also investigate the *in vitro* kinetics of Ca and P uptake onto these glasses, as well as Ca and Si release from their surfaces in order to evaluate their bioactivity and potential efficacy in bone regeneration applications. In our previous work, we quantitatively described the uptake kinetics of Ca and P as well as Si release from different biomaterials including non-doped bioactive glasses;^10,20–22^ however, the ion-exchange process was not considered since our time measurements did not include the initial ion-exchange period. In this study, we quantitatively describe the kinetics of the ion exchange release of Ca from the prepared bioactive glass using a novel approach that can be, as well, applied to similar Ca containing biomaterials. We also investigate the *in vitro* cellular proliferation in BG, silver-doped BG (BG-Ag) and gold-doped BG (BG-Au) nanohybrids using MG-63 cell lines as a representative test model for human osteoblast cell lines.

## 2 Results and discussion

### 2.1 Characterization of the as-prepared nanoparticles and nanocomposites

UV-Vis absorption spectra of the as-prepared AgNPs and AuNPs exhibited a single narrow absorption band at *λ* = 525 and 412 nm, respectively (Fig. S1†), confirming the formation of the nanoparticles. The TEM micrographs (Fig. S2†) show that all prepared particles are almost spherical-like in shape. The average particle size (*d*, nm) of AgNPs is around 15 ± 5 nm (Fig. S2a†), while that of AuNPs is around 20 ± 5 nm (Fig. S2b†).

**Fig. 1:**
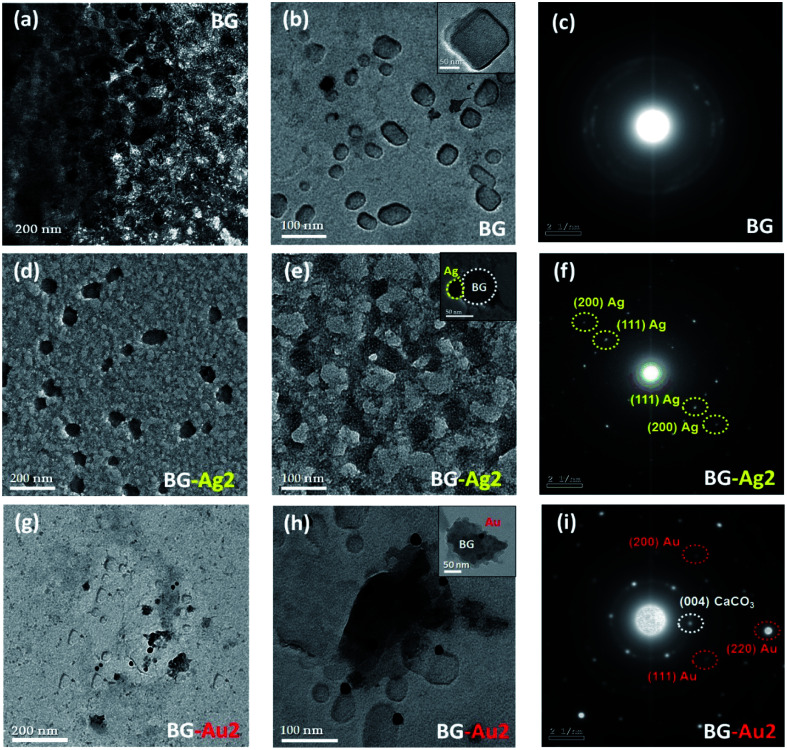
TEM micrographs for BG (a and b), BG-Ag2 (d, e), and BG-Au2 (g, h) at different magnification powers, along with SAED images for BG (c), BG-Ag2 (f), and BG-Au2 (i).

The TEM micrographs and the selected area electron diffraction (SAED) patterns are shown in Fig. 1. Upon doping the BG with AgNPs and AuNPs forming BG-Ag2 and BG-Au2, the respective average particle sizes were 90 ± 20 nm and 150 ± 50 nm (Fig. 1d, e, g and h), as opposed to 100 ± 50 nm for BG (Fig. 1a and b). This implies that incorporating the NPs did not significantly affect the size of BG. It was reported in previous literature that doping bioactive glass with silver did not affect its particle size.^4,23^ In addition, no diffraction rings were observed in the SAED patterns of BG which confirms its amorphous nature (Fig. 1c). However, upon incorporating AgNPs and AuNPs into BG, diffraction rings appeared as shown in Fig. 1f and i, respectively. This indicates the poly-crystalline structures of BG-Ag and BG-Au. The presence of a metallic silver crystalline phase in silver-doped bioglass was previously reported.^6,24^

**Fig. 2:**
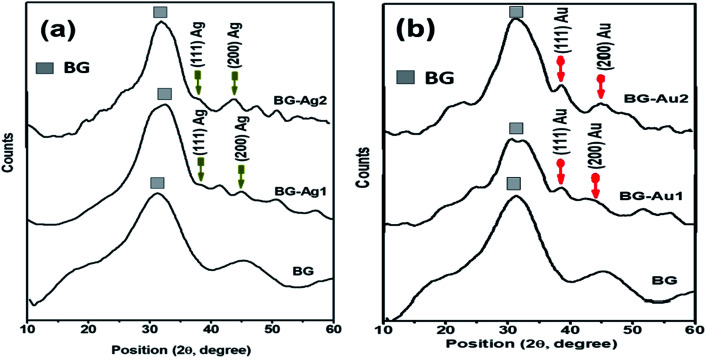
XRD patterns for BG, BG-Ag (a), and BG-Au (b) nanocomposites.

Characterization of BG and its hybrid nanocomposites with metallic nanoparticles (*i.e.*, Ag and Au NPs) by X-ray diffraction analysis revealed an amorphous structure for BG (Fig. 2a and b), and a crystalline structure after incorporating AgNPs and AuNPs. This finding supports the SAED results discussed earlier. However, the crystalline structure was less pronounced at the lowest concentration (BG-Au1 and BG-Ag1) which was particularly selected since it showed no cytotoxic effect when used in composites, as was previously reported.^20,21^ The amorphous structure of bioactive glass, as a major constituent of the composites, camouflaged the crystalline and the characteristic peaks; especially the (111) reflection plan, in each of Ag and Au NPs which represent minor phases in the composites.

The colloidal properties based on DLS and zeta potential measurements for BG and its nanocomposites are presented in Table 1, where it is clear that the hydrodynamic particle size of BG-Ag nanocomposites decreased relative to pure BG. By increasing the concentration of AgNPs in the nanocomposites, they showed excellent dispersion as evident from the low PDI (∼0.262) of BG-Ag2. Zeta potential of the nanocomposites, on the other hand, slightly increased relative to that of BG probably due to the adhesion of Ag^*n*+^ onto its surface. The release of Ag ions from the surface of Ag-doped bioglasses was previously reported.^6,15^ Following the same trend, the particle size of BG decreased upon the incorporation of AuNPs, while its zeta potential increased probably due to the adhesion of Au^*n*+^ onto its surface which rendered it less negative.

**Table 1:** DLS & zeta potential measurements for bioglass and metal-bioglass nanocomposites

Sample ID	Dynamic light scattering (DLS)		Zeta potential (*ξ*, mV)
	*H*_D_ (nm)	PDI	
BG	**626 ± 70.51**	**0.899**	**−28.9 ± 7.64**
BG-Ag1	33.16 ± 3.655	0.853	−21.7 ± 7.55
BG-Ag2	85.81 ± 12.77	0.262	−24.5 ± 4.69
BG-Au1	59.19 ± 10.03	0.789	−12.6 ± 4.85
BG-Au2	92.54 ± 13.80	0.789	−16.5 ± 5.81

In addition, BG with the higher concentration of Ag NPs (BG-Ag2) exhibited the highest zeta potential (highest negative charge) among the investigated silver and gold nanocomposites. Thus, they became more uniformly dispersed in the solution (*i.e.*, low PDI) due to the repulsive forces between the particles. This led to ‘electrostatic’ colloidal stability and mitigated aggregation. In addition, the higher reactivity of Ag NPs relative to Au NPs could have allowed them to be more uniformly distributed into the BG matrix as shown in the TEM images (Fig. 1). This would lead to more uniform charge distribution and lesser possibility for aggregation. The hydrodynamic sizes of BG-Ag2 and BG-Au2 are comparable to their counterparts measured by TEM, which is not the case for BG whose hydrodynamic size is much larger than its TEM size probably due to its larger hydrated sphere formed by virtue of its larger negative charge or possibly higher charge density relative to the nanocomposites.

The following discussion will be concerned with investigating the *in vitro* kinetics of Ca release, Ca and P uptake as well as Si release from BG and its nanocomposites (BG-Ag and BG-Au). First, the kinetic study pertaining to the glass doped with silver nanoparticles will be presented, followed by the study pertaining to the glass doped with gold nanoparticles. These kinetic studies will be complemented with cell culture studies to investigate the cytotoxic effects and proliferative impact of BG and its nanocomposites on human bone cells.

### 2.2 Doping with silver nanoparticles

#### 2.2.1 Ca release

**Fig. 3:**
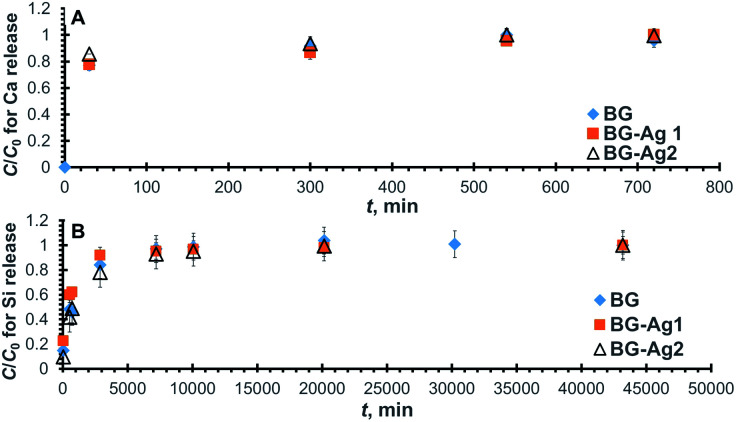
Normalized concentration profiles for the release of Ca (A) and Si (B) from BG-Ag after soaking in SBF.

Fig. 3A depicts the normalized concentration profiles for the release of Ca from the bioactive glass doped with 0, 0.006, and 0.01 mg l^−1^ of silver nanoparticles, referred to as BG, BG-Ag1, and BG-Ag2, respectively. Clearly, the normalized concentration of Ca ions in SBF increases with time until it reaches a constant equilibrium value at unity for all tested bioactive glasses at about 600–800 min (10–13 h).

The increase in Ca release with time during 3 immersion days was previously reported for silver-doped mesoporous bioglasses with a composition of 78% SiO_2_, 20% CaO, 0.8% Ag_2_O, and 1.2% P_2_O_5_.^25^ As also shown in the figure, the maximum release concentration corresponds to a normalized concentration of unity at the plateau of each profile, and it represents the maximum release capacity.

To quantitatively determine the release capacities and release rates for the prepared bioactive glass composites, the concentration profiles for BG, BG-Ag1, and BG-Ag2 were fitted to the pseudo-second-order kinetic model. In Fig. S3A,† the predicted values from the linearized form of the model equation (eqn (1), Experimental section) are presented as dotted lines while the corresponding experimental measured values are shown as scattered points. The values of the correlation factor (*R*^2^) for the predicted profiles of the three bioactive glasses given in Table 2 indicate a perfect fit.

**Table 2:** Kinetic parameters for Ca and Si release from bare bioactive glass and bioactive glass doped with different concentrations of silver nanoparticles^a^

		BG	BG-Ag1	BG-Ag2
Ca release	*C*_e_, mg l^−1^	156.25	181.82	178.57
	*k* × 10^3^, L mg^−1^ min^−1^	0.62	0.21	0.48
	*ν*_i_, mg L^−1^ min^−1^	15.14	6.942	15.31
	*R* ^2^	0.997	0.995	0.999
Si release	*C*_e_, mg l^−1^	42.37	36.63	37.59
	*k* × 10^3^, L mg^−1^ min^−1^	0.056	0.079	0.059
	*ν*_i_, mg L^−1^ min^−1^	0.101	0.106	0.083
	*R* ^2^	0.999	0.999	0.999

a Values given in the table are average values for triplicates with a maximum % error of 5%.

The relevant kinetic parameters, *C*_e_ (equilibrium concentration) and *k* (rate constant), are also given in the table from which it can be deduced that the release capacity was augmented by about 15% when Ag nanoparticles were added to the bioactive glass. This effect could be either due to alteration in the surface morphology from amorphous for BG to poly-crystalline for BG-Ag as detected by TEM and SAED measurements, or the presence of released Ag ions which could have competed with Ca ions on the adsorption sites. Particle size is not an influencing factor since it was not significantly affected by doping as confirmed using TEM. The kinetic constants, on the other hand, decreased by about 66% and 23%, respectively on the addition of 0.006 mg l^−1^ and 0.01 mg l^−1^ of Ag nanoparticles. The presence of Ag ions in solution could have built up a positive charge thus hindering the mass transfer of Ca ions from the bioactive glass to the solution and, in turn, reducing the rate of release. Although the addition of 0.006 mg l^−1^ of Ag nanoparticles enhanced Ca release capacity and reduced its overall release rate, yet increasing the concentration of Ag nanoparticles from 0.006 mg l^−1^ to 0.01 mg l^−1^ did not significantly change the release capacity but increased the release rate. The high concentration of Ag nanoparticles could have caused their aggregation and/or mitigated the formation of the positively-charged Ag layer, thus increasing the rate of Ca release. As for the initial rates, *ν*_i_ (Table 2), they exhibited a trend similar to that of the overall rate where the rates pertaining to BG and BG-Ag2 are comparable and amount to almost twice that of BG-Ag1.

Similarly, the normalized concentration profiles for Si release from the three bioactive glasses are shown in Fig. 3B and their corresponding linear plots are presented in Fig. S3B.† As in case of Ca release, Si release increases with time until it flattens out after 5–7 days. Similar behaviour was reported in a previous study on silver-doped mesoporous bioglasses with a composition of 78% SiO_2_, 20% CaO, 0.8% Ag_2_O, and 1.2% P_2_O_5_,^25^ however, the study was conducted for only 3 days. The kinetic parameters pertaining to Si release are compiled in Table 2, where it can be deduced that the addition of Ag nanoparticles reduced Si release capacity, although it enhanced Ca release as shown earlier. The presence of nanoparticles could have mitigated the attack of OH groups on the Si–O–Si bond and hence reduced the release of soluble Si in solution. Furthermore, the addition of 0.006 mg l^−1^ of Ag nanoparticles raised the kinetic rate constant by about 40%, but it dropped to almost its original value by the addition of 0.01 mg l^−1^ nanoparticles. On the other hand, the presence of nanoparticles did not increase the initial rate.

#### 2.2.2 Ca and P uptake

**Fig. 4:**
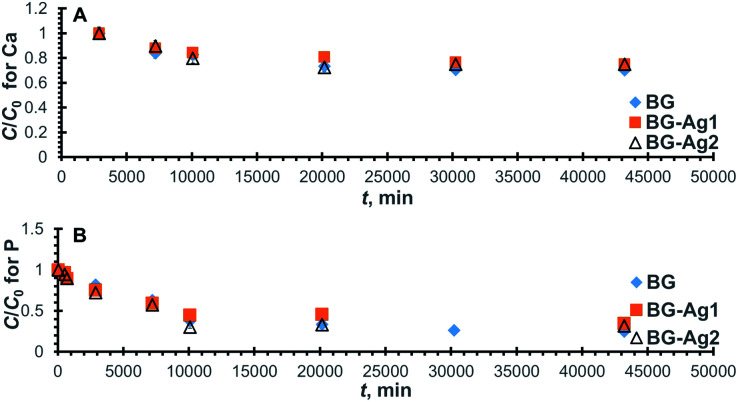
Normalized concentration profiles for Ca (A) and P (B) ions remaining in SBF after uptake onto BG-Ag.

The ion exchange step that involved Ca release lasted up till 720 min (Fig. 3A), then the concentration of Ca was kept constant, after which it dropped at about 2500 min (Fig. 4A), indicating the uptake of Ca onto the bioactive glass. The uptake of P, on the other hand, started from the very beginning since there is no P release. The normalized concentration profiles for Ca and P onto the employed bioactive glasses are shown in Fig. 4A and B, respectively. For all profiles, it can be observed that the normalized concentration and hence, the concentration decreases with time until it reaches a fixed value at equilibrium where the profiles flatten out. The value of the concentration thereat corresponds to the equilibrium uptake capacity of the bioactive glass (*q*_e_). For Ca (Fig. 4A), it is obvious that the uptake capacities onto all the employed bioactive glasses are comparable since all Ca profiles reach almost the same constant normalized concentration. As for P (Fig. 4B), there is a slight decrease in uptake with the addition of silver nanoparticles. Furthermore, all profiles reached equilibrium after about 30 000 min (21 days) when approximately 30% and 70%, respectively of Ca and P in solution have been adsorbed onto the bioactive glass.

The relevant linear plots for the pseudo-second-order model were constructed similar to those shown in Fig. S3† and predicted values were in good agreement with the experimental measurements as can be deduced from the high values of *R*^2^ (Table 3). The uptake capacity (*q*_e_) and second-order rate constant (*k*) for the sorption of Ca and P onto the different examined bioactive glasses were retrieved from the linear plots and are given in Table 3. Clearly, there was no significant improvement in Ca uptake capacity upon the addition of Ag nanoparticles to the bioactive glass. The adsorption of Ca is a consequence of Ca release and, in turn, Si release. Although Ca release capacity increased with the addition of nanoparticles, yet its uptake remained unchanged probably as a result of the reduced Si release capacity. Furthermore, almost a 20% increase in the kinetic rate constant, and the initial rate was achieved using 0.01 mg l^−1^ concentration of Ag nanoparticles.

**Table 3:** Kinetic parameters for Ca and P uptake onto bare bioactive glass and bioactive glass doped with different concentrations of silver nanoparticles^a^

		BG	BG-Ag1	BG-Ag2
Ca uptake	*q*_e_, mg g^−1^	107.53	105.26	109.89
	*k* × 10^7^, g mg^−1^ min^−1^	9.46	8.62	11.08
	*ν*_i_, mg g^−1^ min^−1^	0.011	0.010	0.013
	*R* ^2^	0.999	0.995	0.920
P uptake	*q*_e_, mg g^−1^	62.11	52.36	59.88
	*k* × 10^6^, g mg^−1^ min^−1^	2.11	3.98	3.50
	*ν*_i_, mg g^−1^ min^−1^	0.008	0.011	0.013
	*R* ^2^	0.986	0.993	0.984

a Values given in the table are average values for triplicates with a maximum % error of 5%.

As for P, the uptake capacity was not enhanced with the addition of Ag nanoparticles. However, the rate constant values were augmented by about twice and 1.7 times and the initial rates were increased by 1.4 and 1.6 times when BG-Ag1 and BG-Ag2 were used, respectively. The presence of nanoparticles changed the morphology of the bioactive glass as indicated earlier, thus creating new surfaces with more vacant active sites. This, in turn, would enhance the rate of uptake.

### 2.3 Doping with gold nanoparticles

Following the same line of reasoning adopted with bioactive glasses doped with silver nanoparticles, a kinetic study was conducted on bioactive glasses doped with gold nanoparticles. The kinetics of Ca and Si release, as well as Ca and P uptake onto these bioactive glasses, were studied.

#### 2.3.1 Ca and Si release

**Fig. 5:**
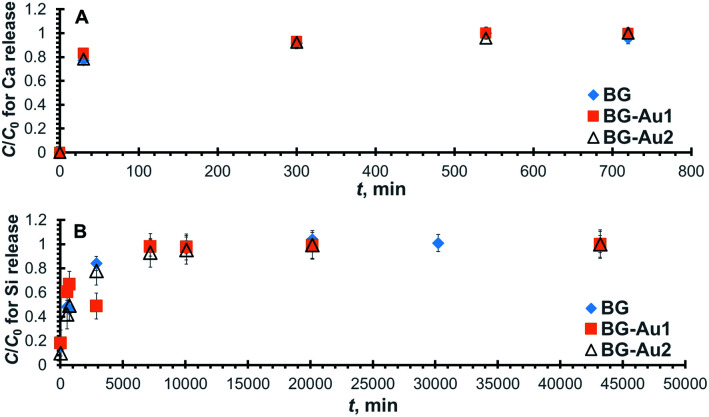
Normalized concentration profiles for the release of Ca (A) and Si (B) from BG-Au after soaking in SBF.

The normalized concentration profiles for Ca and Si release from BG, BG-Au1, and BG-Au2 are depicted in Fig. 5A and B, respectively, while their relevant pseudo-second-order linear plots are shown in Fig. S4A and B,† respectively. The kinetic parameters obtained from these plots are compiled in Table 4, where it can be deduced that the nanogold-doped bioactive glasses have higher Ca release capacities and lower release rate constants than the non-doped bioactive glass. This was also the case for nanosilver-doped bioactive glasses. Such behaviour could be owed to the release of Au^*n*+^ ions in solution, which could have competed with Ca^2+^ ions on the adsorption sites. Furthermore, the addition of gold nanoparticles reduced the release rate constant and initial release rate for Si, as well as the release capacity. This could be attributed to reasons alluded to earlier when discussing the release of Si from silver-doped bioactive glasses. The effect of nanoparticle concentration on the release capacity trend is similar to that encountered with silver-doped bioactive glasses. In general, increasing the concentration of gold nanoparticles did not show a substantial effect on Ca and Si release capacities or their release rates.

**Table 4:** Kinetic parameters for Ca and Si release from bare bioactive glass and bioactive glass doped with different concentrations of gold nanoparticles^a^

		BG	BG-Au1	BG-Au2
Ca release	*C*_e_, mg l^−1^	156.25	178.57	175.44
	*k* × 10^3^, L mg^−1^ min^−1^	0.62	0.42	0.33
	*ν*_i_, mg L^−1^ min^−1^	15.14	13.39	10.16
	*R* ^2^	0.997	0.999	0.998
Si release	*C*_e_, mg l^−1^	42.37	35.84	34.72
	*k* × 10^3^, L mg^−1^ min^−1^	0.056	0.036	0.044
	*ν*_i_, mg L^−1^ min^−1^	0.101	0.046	0.053
	*R* ^2^	0.999	0.996	0.999

a Values given in the table are average values for triplicates with a maximum % error of 5%.

#### 2.3.2 Ca and P uptake

**Fig. 6:**
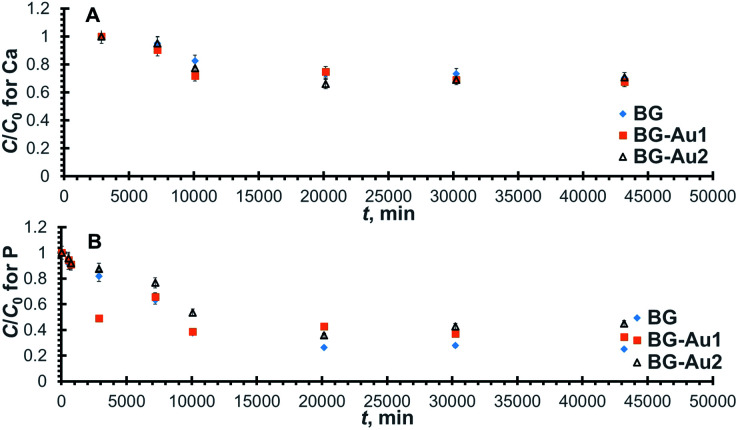
Normalized concentration profiles for Ca (A) and P (B) uptake onto BG-Au after soaking in SBF.

The uptake profiles for Ca and P onto the investigated bioactive glasses are presented in Fig. 6A and B, respectively. As can be observed, all Ca profiles reach equilibrium at about 2000 min (14 days), where approximately 30% of Ca in the solution has been taken up by the bioactive glass. For P, all profiles reach equilibrium at about the same time as Ca profiles, with bioactive glass adsorbing approximately 60% to 70% of P in solution depending on the concentration of gold nanoparticles present in the bioactive glass.

The kinetic parameters were derived from the linearized forms of the pseudo-second-order equations pertaining to the profiles in Fig. 6. By inspecting the values of these parameters (Table 5), it can be inferred that the addition of gold nanoparticles enhanced Ca uptake capacity but did not improve P uptake. Unlike the case of silver nanoparticles, the uptake of Ca was improved probably due to the lower reactivity of gold as compared to silver nanoparticles, which could have led to less competition over the active sites. The presence of gold nanoparticles also increased the rate constant and, consequently, the initial rate for both Ca and P uptake. This behaviour is similar to that encountered with silver nanoparticles.

**Table 5:** Kinetic parameters for Ca and P uptake onto bare bioactive glass and bioactive glass doped with different concentrations of gold nanoparticles^a^

		BG	BG-Au1	BG-Au2
Ca uptake	*q*_e_, mg g^−1^	107.53	128.21	109.89
	*k* × 10^7^, g mg^−1^ min^−1^	9.46	16.0	61.0
	*ν*_i_, mg g^−1^ min^−1^	0.011	0.026	0.074
	*R* ^2^	0.999	0.971	0.970
P uptake	*q*_e_, mg g^−1^	62.11	54.64	49.50
	*k* × 10^6^, g mg^−1^ min^−1^	2.11	3.95	2.36
	*ν*_i_, mg g^−1^ min^−1^	0.008	0.012	0.006
	*R* ^2^	0.986	0.980	0.924

a Values given in the table are average values for triplicates with a maximum % error of 5%.

### 2.4 Comparative analysis

To select the bioactive glass appropriate for a specific application, the % increase or decrease in Ca and P uptake capacities, rate constants and initial rates for the doped bioactive glasses relative to their non-doped counterparts were compiled in Table 6. In general, all silver doped bioactive glasses enhanced the rate of P uptake but decreased the uptake capacity. As for Ca, only BG-Ag2 was successful in improving the rate while almost maintaining the original capacity. Doping with gold nanoparticles, on the other hand, was beneficial in enhancing Ca and P uptake rates as well as Ca uptake capacity.

**Table 6:** Percentage increase or decrease in kinetic parameters for Ca and P uptake onto doped bioactive glasses relative to the non-doped one^a^

		BG-Ag1	BG-Ag2	BG-Au1	BG-Au2
Ca uptake	% Δ*q*_e_	−2.110	+2.190	+19.23	+2.190
	% Δ*k*	−8.880	+17.12	+69.13	+544.8
	% Δ*ν*_i_	−9.091	+18.18	+136.4	+572.7
P uptake	% Δ*q*_e_	−15.70	−3.590	−12.03	−20.30
	% Δ*k*	+88.63	+65.88	+87.20	+11.85
	% Δ*ν*_i_	+37.50	+62.50	+37.50	+962.5

a Values of less than 5% are non-significant since the maximum % error is 5%.

Among the four doped bioactive glasses, BG-Au1 is the most favourable for Ca uptake capacity as it enhanced the uptake by about 19% relative to BG. BG-Au2, on the other hand, is the most recommended for Ca uptake rate since it augmented the rate by about 7 times relative to BG. Both BG-Ag1 and BG-Au1 could be favoured for P uptake rate as they increased the uptake by about 88% over that of BG. No doped bioactive glass, however, was successful in enhancing P uptake capacity.

### 2.5 Human bone cell proliferation

**Fig. 7:**
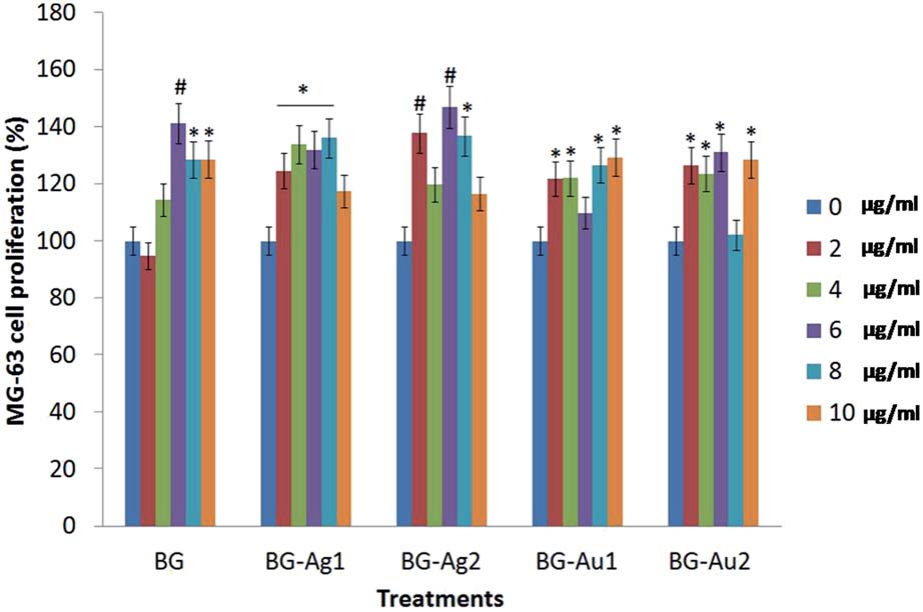
Cell viability determined using MTT assay after treatment of MG-63 cell line with serial concentrations (0, 2, 4, 6, 8 and 10 μg ml^−1^) of proposed treatments. Data were measured after 24 h. (*) means significant difference (*P* < 0.05) compared to control, (#) means high significant difference (*P* < 0.01) compared to control.

The cell proliferation (%) profiles of the osteosarcoma MG-63 cells upon treatment with different concentrations of BG, BG-Ag1, BG-Ag2, BG-Au1, and BG-Au2 are shown in Fig. 7 and Table S1.† Recently, MG-63 cell line has become a standard model, as primary human osteoblasts, for testing biomaterials in cell proliferation studies,^26–32^ being capable of producing action potentials as with primary osteoblasts.^33^ Clearly, all formulations of BG, BG-Ag1, BG-Ag2, BG-Au1, and BG-Au2 induced the proliferation of the MG-63 cells in different patterns. These formulations were used in low dosages (0, 2, 4, 6, 8, and 10 μg ml^−1^), suggesting that the elevation pattern in the cell proliferation may occur even at low concentration. Most of the applied concentrations enhanced the proliferation relative to the control, while very few concentrations showed no significant effect. Upon applying different concentrations of BG, the proliferation (%) of MG-63 cells significantly increased relative to the control recording 141.25% at a dosage of 6 μg ml^−1^. Even though the proliferation declined significantly to 128.40% and 128.61% at dosages of 8 and 10 μg ml^−1^, respectively, yet it remained significantly higher than the control. These results demonstrated the good compatibility of the BG and metal-doped BG nanohybrids with the MG63 cells, and their non-cytotoxic effect on these cells.

When BG-Ag1 was applied at different concentrations, the proliferation (%) of MG-63 cells significantly increased compared to the control reaching 136.06% at a dosage of 8 μg ml^−1^. However, the proliferation (%) decreased to 117.37% at 10 μg ml^−1^ to record insignificant change relative to the control. With BG-Ag2, on the other hand, the proliferation (%) of MG-63 cells showed its maximum significant increase relative to the control at 146.91% when 6 μg ml^−1^ dosage was applied. At a dosage of 8 μg ml^−1^, the proliferation decreased to 136.70% which is significantly higher than the control. At 10 μg ml^−1^, however, the proliferation (116.46%) was insignificant relative to the control.

Upon the application of 2, 4, 8 and 10 μg ml^−1^ of BG-Au1, the proliferation (%) of MG-63 cells significantly increased compared to the control recording its maximum value of 129.23% at 10 μg ml^−1^. With BG-Au2, on the other hand, the highest significant proliferation (%) of MG-63 cells was recorded at 131.12% when 6 μg ml^−1^ dosage was applied. Other applied dosages of 2, 4 and 10 μg ml^−1^ were also successful in significantly enhancing the proliferation (%) recording comparable values to that obtained with 6 μg ml^−1^. The above results confirm the biocompatibility of all prepared nanocomposites. They also demonstrate the stimulating effect of these nano-composites on MG-63 cells when applied at a low concentration range of 2–10 μg ml^−1^.

The above results are in agreement with previous reports that dealt with the enhancement in the cellular proliferation percentage upon treatment with BG, AuNPs and AgNPs.^34,35^ Mârza *et al.* reported that BG has the ability to promote the growth of granulation tissue. In addition, AuNPs accelerated the healing of wounds by possibly inducing angiogenesis, tissue regeneration and connective tissue formation.^34^ In case of BG-Ag nanohybrids, Xie *et al.* reported in their study that exposure to low concentrations of AgNPs shortly after treatment induced cell proliferation.^35^ This effect was owed to the induction of hormesis,^36,37^ which enhances cell viability and stimulates a compensatory or an adaptive response behaviour in organisms.

Our experimental results using MTT assay reflected the remarkable induced cellular proliferation upon the treatment of MG-63 cell lines with BG, BG-Ag and BG-Au nanohybrids at different low doses of 2, 4, 6, 8 and 10 μg ml^−1^. In the MTT assay, the conversion of 3-[4,5-dimethylthiazole-2-yl]-2,5-diphenyltetrazolium bromide (MTT) to MTT-formazan is catalysed by the mitochondrial succinate dehydrogenase (reductase),^38^ as reported in previous studies.^30,31,35,39–45^ It was therein suggested that the treatment of the MG-63 cell lines with BG stimulates osteogenesis^31^ by means of the mitogen-activated protein kinases (MAPKs)^42^ that play an essential role in the genetic activation of MG-63 cells, thus enhancing the synthesis of MG-63 DNAs, which, in turn, stimulates the cellular division of MG-63.^40^ Thus, the cells show better initial attachment to the rough surface of BG.^39^ Furthermore, changes in the particle surface chemistry also contribute to controlling the bone-forming cell behaviour by generating a substrate on the particle surface that drives such cells to proliferate, in a manner similar to bone.^40^ Moreover, it was reported that the presence of AuNPs did not alter the cellular morphology and apoptosis rate or induce cellular necrosis.^30^ Even at low concentrations, AuNPs promoted the cellular differentiation and proliferation.^46^ Finally, and with regard to our MTT proliferation results, the proliferation rate in BG-Au is lower than that in BG-Ag nanohybrids. This finding is in agreement with that of Uboldi *et al.* who reported that the presence of citrate ions on the surface of AuNPs leads to reduction in the cellular viability and proliferation.^47^ Other reports indicated that AgNPs could increase the cellular proliferation even at low concentrations due to hormesis.^35^

**Fig. 8:**
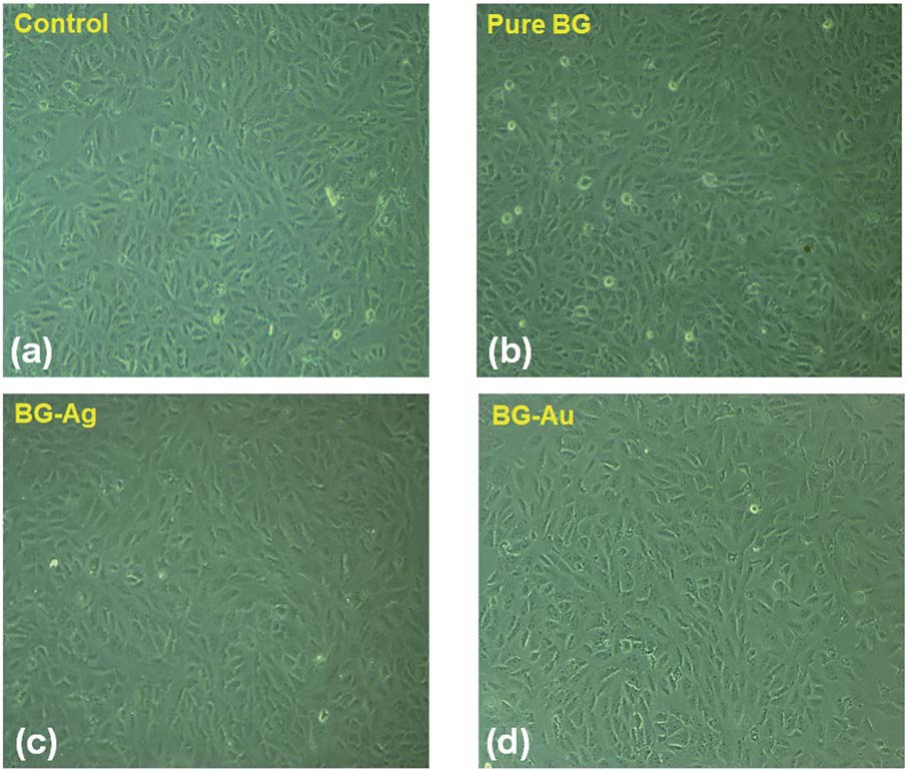
Inverted microscopy investigation for cellular morphology of (a) untreated MG-63 cell lines, and cells treated with (b) bare BG, (c) BG-Ag and (d) BG-Au nanohybrids at a dose of 6 μg ml^−1^.

Furthermore, inverted microscopy investigation was performed to confirm the cellular proliferation upon the treatment of MG-63 cell lines with BG, BG-Ag and BG-Au nanohybrids with a dose of 6 μg ml^−1^ (Fig. 8). A clear enhancement in the cellular proliferation of osteoblast cell lines upon treatment with all of these nanohybrids can be observed, as compared to the control (Fig. 8). This is consistent with the MTT assay results shown earlier in Fig. 7. In addition, no significant morphological differences can be observed between the cells treated with BG, BG-Ag or BG-Au (Fig. 8). Besides, the number of apoptotic cells upon treatment with BG was higher than its counterpart for BG-Ag, which, in turn, was higher than that for BG-Au nanohybrids (Fig. 8).

## 3 Experimental

### 3.1 Synthesis and characterization of the bioactive glass composite

Bioactive glass 46S6 of the composition (46% SiO_2_, 24% CaO, 24% Na_2_O, 6% P_2_O_5_ by mol) was prepared through the melt quench route as previously described.^4,22^ Materials used were trisodium tri-metaphosphate (Na_3_P_3_O_9_, Sigma-Aldrich, US), sodium metasilicate pentahydrate (Na_2_SiO_2_(OH)_2_·4H_2_O, Sigma-Aldrich, US) and calcium silicate (CaSiO_3_, Alfa Aesar, Germany). The glass powder was melted in a Pt 2% Rh crucible covered with Pt foil to minimize phosphorus evaporation, and was placed in an electric furnace at 1300–1350 °C for 2 h. The melt was then homogenized by swirling, and a portion of which was cast into rods and button-like shapes. These were then adequately annealed at 450 °C in a muffle furnace to minimize residual stresses. The produced bulk glasses were crushed and sieved to obtain glassy particles of less than 40 μm.

Gold (AuNPs) and silver (AgNPs) nanoparticles were synthesized *via* chemical reduction. Typically, AgNPs were prepared by the addition of about 1.4 mM of the silver ion solution (AgNO_3_, Sigma-Aldrich, US) to an aqueous solution of 1% w/v of trisodium citrate (Alfa Aesar, Germany), along with polyvinyl pyrrolidone (PVP 40k, Sigma-Aldrich, US). The mixture was vigorously stirred and a few drops of freshly prepared ice-cooled sodium borohydride (NaBH_4_, Win-Lab, UK) solution of 0.1 M were added.^20,48,49^ In this method, NaBH_4_ acts as the reducing agent, while tri-sodium citrate and PVP serve as capping materials which prevent particle aggregation and further growth. As for AuNPs, they were prepared *via* chemical reduction of gold ions (Au^3+^) in the presence of trisodium citrate as a capping and reducing agent according to the method proposed by Mostafa *et al.*^21^ and Shaat *et al.*^50^ Typically, a 90 ml aqueous solution of 5 mM of gold ions (HAuCl_4_·H_2_O, Sigma, US) was added to 1 g of polyvinyl alcohol (PVA, Alfa-Aesar, Germany) under vigorous stirring and boiling. Afterward, a volume of 10 ml of 0.05 M of trisodium citrate was added, causing the colour of the solution to change from pale yellow to deep red. At this point, the solution was stirred for an additional 10 min and was left to cool at room temperature. The optical properties of the as-prepared metallic nanoparticles were investigated *via* UV-Vis absorption spectroscopy using TG80 instrument within the range of 350–900 nm, with a slit width of 0.2 nm. The morphological properties were also explored *via* Transmission Electron Microscopy (TEM) imaging using JEOL 2100 LB_6_ operated at 120 kV.

Metal-bioglass nanocomposites were prepared by gently mixing either AgNPs or AuNPs with 1 g of the prepared bioactive glass composite. Two concentrations of 0.006 and 0.01 ppm were used for the AgNPs yielding BG-Ag1 and BG-Ag2 composites, respectively and the same concentrations were also employed for AuNPs to obtain BG-Au1 and BG-Au2 composites, respectively. The prepared mixtures were then left to dry at 50 °C overnight. Structure of the prepared composites was confirmed by X-ray diffraction (XRD) using BRUKER AXS D8 ADVANCE diffractometer with a Cu target (Cu Kα radiation), performed under a 2*θ* range of 15–60°, with a step of 0.2° min^−1^.

### 3.2 *In vitro* kinetics

The *in vitro* kinetic experiment was conducted by immersing 30 mg of each of BG, BG-Ag1, BG-Ag2, BG-Au1 and BG-Au2 powder into 60 ml of SBF that has an ionic composition simulating that of human blood plasma at 37 °C.^51^ Samples were soaked in SBF for different time intervals with shaking at 37 °C and 50 rpm. Concentrations of Ca, P, and Si in the solution were then measured by Inductively Coupled Plasma Atomic Emission Spectroscopy (ICP-AES). According to the mechanism of apatite formation, the initial stage of ion exchange entails the release of Ca. Hence, the concentration of Ca in solution represents Ca release. In the later stages, Ca is adsorbed onto the surface of the bioactive glass composites, and hence its concentration decreases in solution. In addition, P is adsorbed onto the bioactive glass composites while Si is released right upon the immersion of the bioactive glass composite in solution. The amount of adsorbed Ca, and P were calculated by mass balance, as shown in our previous work.^20^

The concentration profiles obtained from the experimental measurements were fitted to the pseudo-second-order kinetic model,^20,21^ the linear form of its equation is given by;1
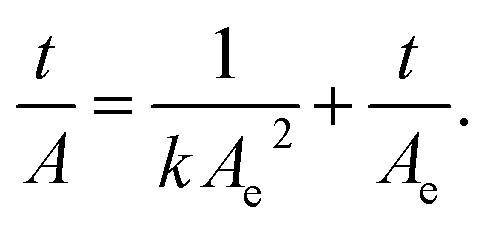
Where *t* is the time of immersion, *k* is the pseudo-second-order rate constant, *A* and *A*_e_ are the amounts adsorbed (mg g^−1^) or released (mg l^−1^) at time *t* and in approach to equilibrium, respectively. The amounts adsorbed onto the glass will be henceforth denoted by *q* (mg g^−1^), whereas the amounts released in solution will be denoted by *C* (mg l^−1^).

Initial rates of adsorption (uptake) or desorption (release) are expressed as follows;

2 *ν*_i_ = *kA*_e_^2^

### 3.3 *In vitro* cell culture studies for the bioactive glasses

#### 3.3.1 Cell culture, maintenance, and sub-culture

In the current study, Human MG-63 osteoblast-like cells purchased from Cell Line Services (CLS, GmbH, Eppenheim, Germany) were cultured with 5% of CO_2_ in Dulbecco's minimum essential medium (Lonza, Switzerland) at 37 °C. The medium was supplemented with 10% fetal bovine serum (Lonza, Switzerland) and 1% penicillin streptomycin (Lonza, Switzerland). Cells were grown for 48 h, until a confluence of about 75% was reached, then they were trypsinized and centrifuged as cell pellets. The pellets were resuspended in fresh medium for subculture. Propagating cells from 75% confluent cultures were harvested and seeded in 96-well plates containing 100 μL of DMEM (10% FBS), at a density of 15 000 cells per well. A cell density number of 1 × 10^4^ cells per well was selected for optimization studies. Finally, the cells were allowed to rest overnight.

#### 3.3.2 Cell proliferation by MTT assay

For each cell line, the percentages of viable cells were measured after treatment with serial dilutions (0, 2, 4, 6, 8 and 10 μg ml^−1^) of the prepared bioglasses (BG, BG-Ag1, BG-Ag2, BG-Au1, BG-Au2). Cell viability was evaluated by the 3-[4,5-methylthiazol-2-yl]-2,5-diphenyl-tetrazolium bromide (MTT) assay as reported elsewhere,^52^ with some modification. After evaluating the cell count and viability by trypan blue dye-based method, 1 × 10^4^ MG-63 cells per well were seeded in a 96-well plate and then left overnight for attachment. On the following day, the medium was completely replaced with a fresh one and different prepared concentrations of the bioglasses were tested on each cell line. In this regard, the cells were incubated for 24 h. In each well, 10 μl of the MTT (5 mg ml^−1^) were added four hours before completion of the incubation period. At the end of the incubation period, 100 μl of dimethyl sulfoxide (DMSO) were added per well to dissolve the formazan crystals. It was left for a duration of 20 min, after which colour development was measured at 450 nm using a Bio-Tek microplate reader.

#### 3.3.3 Statistical analysis

The statistical analysis was performed using IBM SPSS (version 25) with a population at (*P* < 0.05) and (*P* < 0.01) as compared to control. Kinetic studies were conducted in triplicates and linear regression was performed to predict the fitting kinetic models and the relevant kinetic parameters (*n* = 3). Three different cell proliferation replicates were performed (*n* = 3).

## 4 Conclusions

The uptake kinetics for Ca and P onto silver-doped and gold-doped bioactive glasses with different compositions, as well as Ca and Si release from these bioactive glasses, were investigated. Doping with silver nanoparticles increased Ca release capacity but reduced that of Si. Consequently, Ca and P uptake capacities were not enhanced. However, the respective rates of Ca and P uptake were augmented by up to 17% and 88% as a result of the increase in Si release rate and the decrease in Ca release rate. It can, thus, be inferred that there are various factors that can influence the uptake rate and capacity of Ca and P onto the bioactive glass and hence, apatite formation. The combined effect of Ca and Si release, on one hand and the release of silver ions in solution, on the other hand, are important influencing factors. The migration of the competitive Ag ions, being highly diffusive, to and from the solution affects the amount and rate of mass transfer of Ca and P ions.

Furthermore, gold-doped bioactive glasses followed similar trends to those observed with silver-doped ones with regard to augmented Ca and reduced Si release capacities. Although this did not affect P uptake capacity, yet it led to enhancement in Ca uptake capacity by 19%, unlike in case of silver-doped glasses, probably because gold ions are not as competitive as silver due to their low reactivity. While Ca and Si release rates dropped, Ca and P uptake rates were remarkably augmented by up to 7 and 2 times, respectively.

BG and BG doped with Ag or AuNPs showed no cytotoxic effects on osteosarcoma MG-63 cells while it exhibited remarkable cell proliferation even at low concentration. These preliminary findings confirm the biocompatibility of the prepared nano-composites, which is an essential criterion for evaluating the potential of using biomaterials in clinical applications.

## Author contributions

Mostafa, A. A. conceptualization, funding acquisition, project administration, manuscript revision; El-Sayed, M. H. conceptualization, data analysis and curation, manuscript writing and revision; Emam, A. N. performing synthesis and characterization experiments, data analysis and contributing to the writing of the manuscript; Abd-Rabou, A. A. performing cytotoxicity and cell proliferation tests and contributing to the writing of the manuscript; Dawood, R. M. performing cytotoxicity and cell proliferation tests and contributing to the writing of the manuscript; Oudadesse, H. conceptualization, resources, measurements.

## Conflicts of interest

There are no conflicts to declare.

## Supplementary Material

RA-011-D1RA03876A-s001
