# Supplementary material for: Bioactive glass doped with noble metal nanoparticles for bone regeneration: in vitro kinetics and proliferative impact on human bone cell line
Source: RSC Adv. 2021 Jul 25;11(41):25628–38. doi: 10.1039/d1ra03876a (PMC9036971; doi:10.1039/d1ra03876a)
Supplement: RA-011-D1RA03876A-s001 [file RA-011-D1RA03876A-s001.pdf]

Electronic Supplementary Material (ESI) for RSC Advances.
This journal is © The Royal Society of Chemistry 2021

Bioactive glass doped with noble metal nanoparticles for bone regeneration: *in-vitro* kinetics and proliferative impact on human bone cell line

Amany A. Mostafa [a,b*], Mayyada M.H. El-Sayed [c,*], Ahmed N. Emam [a,b], Ahmed A. Abd-Rabou [d,e], Reham M. Dawood [f] and Hassane Oudadesse [g]

[a] Refractories, Ceramics and Building Materials Department (Biomaterials group), National Research Centre (NRC), El Bohouth St., Dokki, 12622 Cairo, Egypt,

[b] Nanomedicine & Tissue Engineering Lab., Medical Research Center of Excellence (MRCE), NRC, Egypt

[c] Chemistry Department, School of Sciences and Engineering, American University in Cairo, AUC Avenue, New Cairo, 11835, Egypt

[d] Hormones Department, Medical Research Division, National Research Centre, Dokki, Giza, Egypt

[e] Stem Cell Lab, Center of Excellence for Advanced Sciences, National Research Centre, Dokki, Giza, Egypt

[f] Department of Microbial Biotechnology, Genetic Engineering Division, National Research Centre, 33 EL Bohouth Street Dokki, Giza, Egypt, 12622

[g] Universite de Rennes 1, UMR CNRS 6226, 263 av. du general Leclerc, 35042 Rennes Cedex, France

***Corresponding author: amani.mostafa@gmail.com, aa.monem@nrc.sci.eg
Mayyada@aucegypt.edu, Mayyadas@gmail.com**

Supplementary information

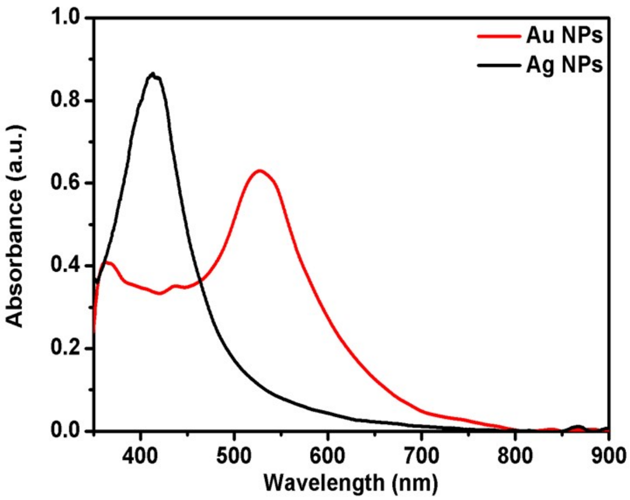

Fig. S1 Normalized absorption spectra for the as-prepared Gold (Au, red line) and (b) Silver (Ag, black line) nanoparticles.

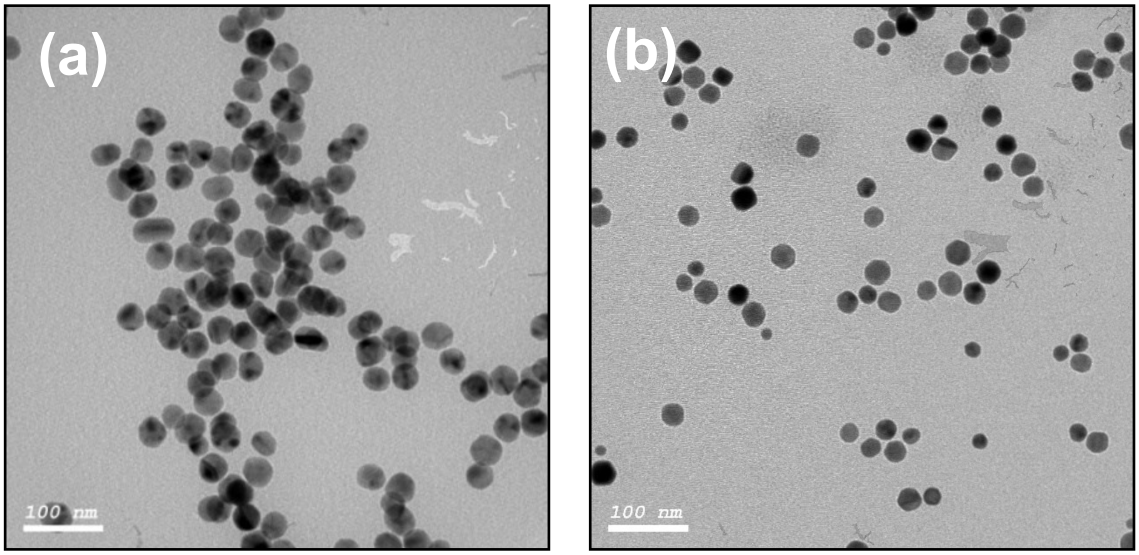

Fig. S2 TEM micrographs for both of as-prepared (a) silver (Ag) and (b) gold (Au) nanoparticles. (Scale bar 100 nm).

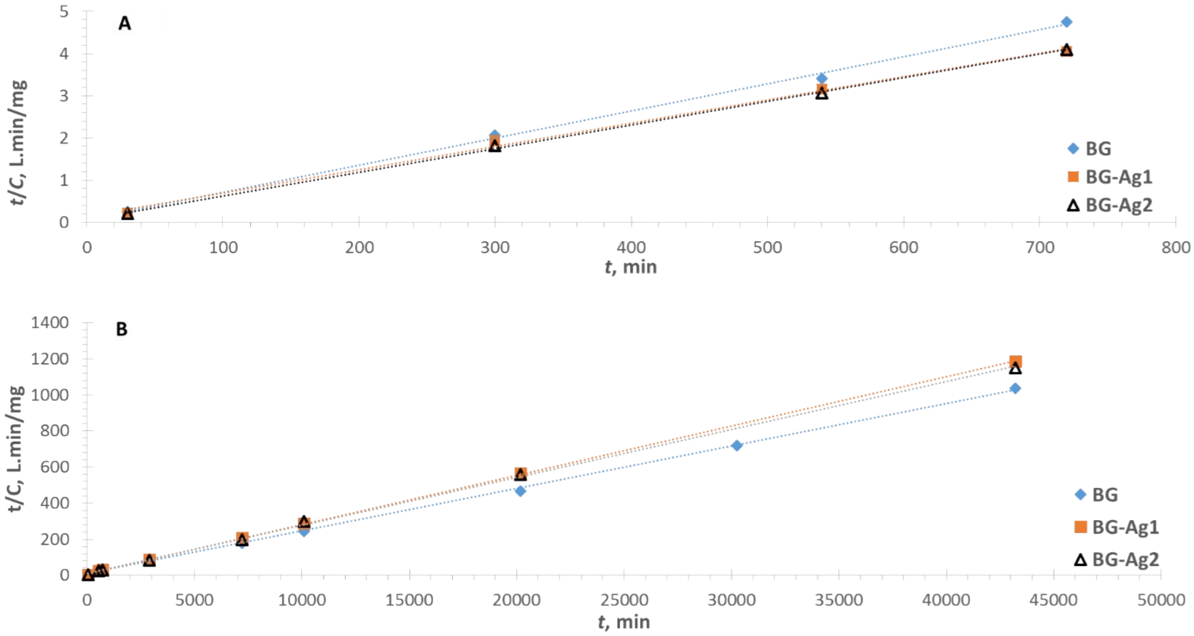

Fig. S3 Linear kinetic plots of the pseudo-second-order kinetic model pertaining to the concentration profiles for the release of Ca (A) and Si (B) from BG-Ag after soaking in SBF.

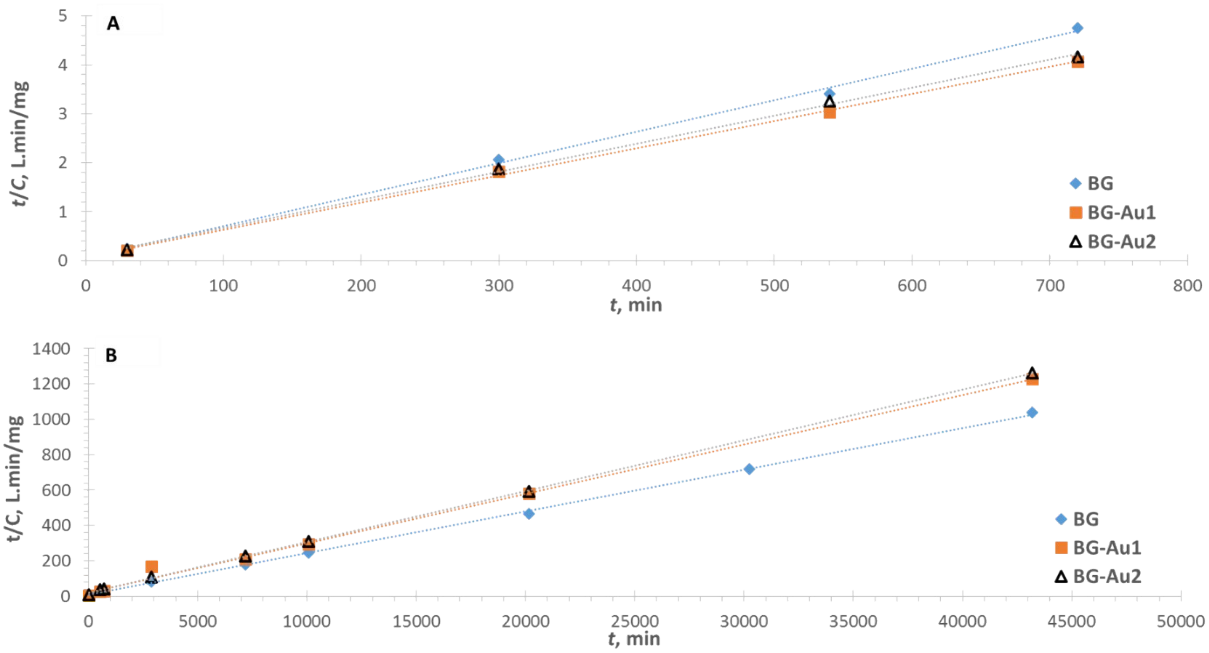

Fig. S4 Linear kinetic plots of the pseudo-second-order kinetic model pertaining to the concentration profiles for the release of Ca (A) and Si (B) from BG-Au after soaking in SBF.

Table S1. Cellular proliferation percentage (%) upon treatment of osteosarcoma MG-63 cell with different doses of BG, and Ag or Au doped BG

Sample<br>Dose (µg/ml)	BG	BG-Ag1	BG-Ag2	BG-Au1	BG-Au2
**0**	100	100	100	100	100
**2**	94.94311	124.511141	137.816245	121.852326	126.496391
**4**	114.243574	133.833561	119.707057	121.937687	123.55763
**6**	**141.255794**	131.969077	**146.915224**	109.773794	**131.120619**
**8**	128.402866	**136.061846**	136.706613	126.547162	102.121959
**10**	128.613569	117.371533	116.466933	**129.236022**	128.354726
